# EANM position paper on article 56 of the Council Directive 2013/59/Euratom (basic safety standards) for nuclear medicine therapy

**DOI:** 10.1007/s00259-020-05038-9

**Published:** 2020-10-15

**Authors:** Mark Konijnenberg, Ken Herrmann, Carsten Kobe, Frederik Verburg, Cecilia Hindorf, Roland Hustinx, Michael Lassmann

**Affiliations:** 1 Department of Radiology and Nuclear Medicine, Erasmus MC, Rotterdam, Netherlands; 2 Department of Nuclear Medicine, University of Duisburg-Essen and German Cancer Consortium (DKTK), University Hospital Essen, Essen, Germany; 3 Department of Nuclear Medicine, Faculty of Medicine and University Hospital Cologne, University of Cologne, Cologne, Germany; 4 Department of Nuclear Medicine Physics, Skåne University Hospital, Lund, Sweden; 5 Service de Médecine Nucléaire et d’Imagerie Oncologique, Département de Physique médicale, Université de Liège - GIGA-CRC in vivo imaging, Centre Hospitalier Universitaire de Liège, Liège, Belgium; 6 Department of Nuclear Medicine, Universitätsklinikum Würzburg, Klinik und Poliklinik für Nuklearmedizin, Oberdürrbacher Str. 6, 97080, Würzburg, Germany

**Keywords:** Nuclear medicine therapy, Dosimetry, Optimization, BSS directive

## Abstract

The EC Directive 2013/59/Euratom states in article 56 that exposures of target volumes in nuclear medicine treatments shall be individually planned and their delivery appropriately verified. The Directive also mentions that medical physics experts should always be appropriately involved in those treatments. Although it is obvious that, in nuclear medicine practice, every nuclear medicine physician and physicist should follow national rules and legislation, the EANM considered it necessary to provide guidance on how to interpret the Directive statements for nuclear medicine treatments.

For this purpose, the EANM proposes to distinguish three levels in compliance to the optimization principle in the directive, inspired by the indication of levels in prescribing, recording and reporting of absorbed doses after radiotherapy defined by the International Commission on Radiation Units and Measurements (ICRU):

- Most nuclear medicine treatments currently applied in Europe are standardized. The minimum requirement for those treatments is ICRU level 1 (“activity-based prescription and patient-averaged dosimetry”), which is defined by administering the activity within 10% of the intended activity, typically according to the package insert or to the respective EANM guidelines, followed by verification of the therapy delivery, if applicable.
- Non-standardized treatments are essentially those in developmental phase or approved radiopharmaceuticals being used off-label with significantly (> 25% more than in the label) higher activities. These treatments should comply with ICRU level 2 (“activity-based prescription and patient-specific dosimetry”), which implies recording and reporting of the absorbed dose to organs at risk and optionally the absorbed dose to treatment regions.
- The EANM strongly encourages to foster research that eventually leads to treatment planning according to ICRU level 3 (“dosimetry-guided patient-specific prescription and verification”), whenever possible and relevant.

Evidence for superiority of therapy prescription on basis of patient-specific dosimetry has not been obtained. However, the authors believe that a better understanding of therapy dosimetry, i.e. how much and where the energy is delivered, and radiobiology, i.e. radiation-related processes in tissues, are keys to the long-term improvement of our treatments.

## Introduction

Personalized therapy was already envisioned by Hippocrates 25 centuries ago: “Let nothing bad be added by the person treating—rather let the evils resulting from the diseases themselves suffice—but only whatever good he is capable of.” [1]. Treatments should be optimized for the individual patient between what is tolerable (add nothing bad or side effects) and whatever good is required for efficacy. In radiotherapy, it is an unquestioned paradigm to perform patient-specific treatment planning prior to any course of treatment with external beams or brachytherapy sources.

This optimization principle has been formalized in the EC Directive 2013/59/Euratom, laying down basic safety standards (BSS) for protection against the dangers arising from exposure to ionizing radiation [2], to be valid for all forms of radiotherapy including nuclear medicine therapy. Specifically, article 56 states:

“For all medical exposure of patients for radiotherapeutic purposes, exposures of target volumes shall be individually planned and their delivery appropriately verified taking into account that doses to non-target volumes and tissues shall be as low as reasonably achievable and consistent with the intended radiotherapeutic purpose of the exposure”.

Nuclear medicine therapy is explicitly included by the definition of “radiotherapeutic” to mean *pertaining to radiotherapy, including nuclear medicine for therapeutic purposes* (condition 81 [2]).

Furthermore, the level of involvement of a medical physics expert is specified in three categories:

(i) In non-standardized therapeutic nuclear medicine practices, a medical physics expert shall be closely involved.
(ii) In standardized therapeutical nuclear medicine practices, a medical physics expert shall be involved.
(iii) For other medical radiological practices, a medical physics expert shall be involved for consultation and advice on matters concerning radiation protection for medical exposure.

The member states had to put into force the laws and regulations necessary to comply with the BSS by at latest 6 February 2018 (cf. Art. 106 of the BSS). Interpretation of the law into practical application is still lacking concerning optimized treatment prescription methods and definitions of standardized and non-standardized therapy. The variations in the practice of nuclear medicine therapies and the related implementation of dosimetry have been explored recently by the EANM internal dosimetry task force [3, 4]. Different approaches to implement the Directive are followed in various European countries, with a good example provided in the consensus document by the Italian nuclear medicine and medical physics associations [5]. Dosimetry-guided treatment is, for most therapeutic compounds, not in line with the instructions for use recommended by European Medicines Agency (EMA), and the posology would need to be modified and complemented in order to comply with the BSS directive. This discrepancy between the optimization principle in the BSS directive and the prescription posology in EMA product approval dossiers for nuclear medicine therapies has been addressed before [6].

## Standardized and non-standardized nuclear medicine therapy

Standardized therapies in nuclear medicine are approved products (by EMA or by CE marking) for radionuclide therapies being administered according to the package inserts or relevant guidelines. Non-standardized therapies are either radionuclide therapies in developmental phase or approved radiopharmaceuticals being used off-label, e.g. by exceeding the maximum allowed activity according to the package insert, also including the total activity administered accumulated over all treatments. The involvement of the medical physics expert (MPE) in standardized therapies entails mostly quality control of the treatment protocol, equipment used and radiation protection responsibility. Recommendations have been formalized by the European Federation of Organizations for Medical Physics (EFOMP) on the involvement of MPEs in radiation therapy; in the practice of standardized therapy, the MPE should monitor this practice regularly and be available on call at all times [7]. We recommend, however, that the MPE is closely involved in the initiation of a new radionuclide therapy for a clinical centre until sufficient routine is gained.

For non-standardized therapies, a medical physics expert should be closely involved meaning to be member of the team that give advice on the therapy for the activity needed (either based on dosimetry or following a fixed activity prescription) and verification of the absorbed doses given in order to avoid acute cases of insufficient dose-coverage or excessive healthy tissue damage. According to the EFOMP [7], an MPE must be present at all times during the entire patient pathway, which does not always seem realistic in clinical practice. However, the MPE does need to be closely involved in the prescription and verification at initial and follow-up non-standardized nuclear medicine therapies.

## Recommendation for levels in compliance to the BSS directive

Evidence for dose-effect relations after radionuclide therapies is in most cases derived from retrospective studies, which are considered to be at higher risk of potential sources of bias and confounding factors as compared with prospective studies [8], and thus hinders the indisputable application of the optimization principle. Clinical evidence for superiority of prospective therapy prescription on basis of patient-specific dosimetry has not been obtained within nuclear medicine therapy. The EANM proposes therefore to distinguish three levels in compliance to the optimization principle in the BSS directive, following the indication of levels in prescribing, recording and reporting of absorbed doses after radiotherapy defined by the International Commission on Radiation Units and Measurements (ICRU) (see, e.g. ICRU report 91, [9]). These three levels are to be viewed as a staircase where above level 1, levels 2 and 3 add refinements, with purpose to decrease the uncertainty in the absorbed dose estimates for the individual patient. Naturally, compliance to level 2 requires that the demands for level 1 are also met and for level 3 that the demands for both levels 1 and 2 are fulfilled.

### Level 1: Activity-based prescription and patient-averaged dosimetry

Level 1 compliance is the minimum requirement for standardized therapies, and below this level, therapy should not be performed. Level 1 is reached by administering a net activity within 10% of the intended activity, typically according to the package insert or to the respective EANM or national guidelines. The net activity is defined as the difference of the activity measured before and after administration. The activity metre (or dose calibrator) should be calibrated for measuring the radionuclide used in the therapy relative to a primary standard issued by a national metrology institute, such that traceability is ascertained, and the amount of activity administered can be accurately determined. Qualitative verification of the therapy delivery should be performed at a relevant time point in therapies for which post-therapy imaging is feasible and the results should be recorded. Absorbed dose estimates can be made for patients involved in level 1 therapies by using patient cohort–averaged dosimetry data and the administered activity. There should be sufficient data available for the practitioner to make an informed decision on the efficacy of delivering the treatment at this level, as the absorbed dose to the target volume is not available at level 1.

### Level 2: Activity-based prescription and patient-specific dosimetry

Level 2 compliance is reached by recording and reporting of the absorbed dose to organs at risk and optionally the absorbed dose to treatment regions (regions of disease that motivate treatment prescription) for the individual patient. This level is advised to form the minimum requirement for non-standardized therapies. If the treatment objective is to avoid toxicity, then the absorbed dose to the organs at risk should be quantified. If the objective is tumour control but also for therapy selection, then the absorbed dose to the treatment region is of relevance. The combined standard uncertainty (one standard deviation) in the relevant absorbed dose is within 20%, depending on the treatment objectives. The activity prescription at level 2 is not different from that in level 1. Level 2 compliance will be useful in deciding on further therapy options at recurrent disease, like external beam radiotherapy or repeating the same therapy as salvage option. Radionuclide therapy in children is highly desired to be given at a level 2 compliance and when possible include optimization to a maximum tolerable absorbed dose in organs at risk (level 3). The organs at risk and treatment regions selected for the calculations need to be those that are most likely to predict biological outcome to assess safety and efficacy of the treatment. Eventually retrospective analysis of the patient-specific dosimetry can lead to an optimized therapy prescription balancing both objectives. The key distinction between level 1 and level 2 is the degree to which the absorbed dose report is patient-specific and also in the degree of its uncertainty. Radiopharmaceuticals in clinical development (phase 1–2 trials) should ideally comply to dose reporting at least at level 2. Likewise, off-label use with administrations of activity that is significantly higher (25% or more) than the recommended activity, including the total activity accumulated over all cycles and treatments, should preferably adhere to level 2.

### Level 3: Dosimetry-guided patient-specific prescription and verification

Level 3 compliance is the prescription of administered activity calculated to deliver a desired absorbed dose to a treatment region or organ at risk and is appropriate in a research setting to develop new dosimetry methodologies in order to better predict response or toxicity. For level 3 studies, it is essential that dosimetry and correlations between absorbed dose and induced effects are timely identified and published in peer-reviewed literature. These studies may lead to an improved characterization of tumour control and normal tissue complication profiles which could produce more accurate dose-effect relationships. Treatment planning according to a personalized dosimetry would fall within level 3, and any uncertainty in the absorbed dose estimates should be reported, including possible non-uniformity of the absorbed dose distribution. Reporting of the clinical dosimetry at level 3 should ideally provide a complete overview of the absorbed dose assessment steps enabling reproducibility and expansion of the results [10].

## Guidance material for treatment and dosimetry protocols

Several guidance documents have been published on recommended dosimetry procedures for standard radionuclide therapies. Basic standards and nomenclature within dosimetry can be found in the MIRD pamphlets 16, 20, 21 and 22 [11–14], and guidelines for quantitative SPECT are provided in MIRD pamphlets 23, 24 and 26 [15–17]. The EANM dosimetry committee has published standard operating procedures for several radionuclide therapies [18–21], and recommendations for dosimetry of ^90^Y liver embolization are currently under evaluation. Additionally, recommendations have been written on reporting clinical dosimetry data and a practical guidance on the quantification of the uncertainties associated with dosimetry [10, 22].

Evidence for superiority of therapy prescription on basis of patient-specific dosimetry has not been obtained, although considering ionizing radiation as its mechanism of action absorbed dose is more likely to form a better measure of outcome. A risk analysis model following, e.g. the Failure Mode and Effects Analysis (FMEA) risk model [23], could form a valuable tool to evaluate whether prescription based on fixed activity and patient average dosimetry could lead to serious events or conversely underdosing in a specific patient cohort leading to sub-optimal outcome.

## Classification of current nuclear medicine therapies

Table 1 indicates radionuclide therapies that are classified as standardized and non-standardized, as well as the prescription basis with indication of the appropriate level. Classified as standardized therapies are those that, to date, have marketing authorization by the EMA for the indication stated in the package insert. According to this classification, all listed types of therapies except [^90^Y]Y-DOTATOC, ^32^P-labelled microparticles, [^177^Lu]Lu-lilotomab satetraxetan and [^177^Lu]Lu-PSMA-ligands are considered standardized therapies at present. Note that this classification does not apply when treatment is given according to a protocol that differs from the approved one, in which case the application is non-standardized and dosimetry should generally be included (L2). Post-therapy dose verification is not always feasible due to the emission spectrum and a diagnostic companion radiopharmaceutical is indicated.

**Table 1:** Classification of radionuclide therapies into standardized and non-standardized modalities with options for prescription and dosimetry, according to the level of compliance to the BSS directive’s optimization principle

Disease	Radionuclide/radiopharmaceutical	Standardized	Prescription (level)	Dosimetry	Guidance report
Benign thyroid disease	[^131^I]NaI	Yes	Activity (L1) or absorbed dose (L3)	Optional	EANM [19, 24]
Differentiated thyroid cancer	[^131^I]NaI	Yes	Activity (L1) or absorbed dose (L3)	Optional	EANM [21, 25] MIRD [16]
Neuroblastoma in children	[^131^I]mIBG	Yes	Activity/BW (L2)/lesion absorbed dose (L3)	Advisable (L2/L3)	EANM [18, 26] MIRD [16]
Neuroendocrine	[^131^I]mIBG	Yes	Activity (L1)	Optional (L2)	EANM [18, 26] MIRD [16]
	[^90^Y]Y-DOTATOC (and other ^177^Lu- or ^90^Y-labelled SSTR ligands)	No	Activity/BSA (L2) or absorbed dose (L3)	Advisable (L2)	EANM [27] MIRD [12]
	[^177^Lu]Lu-DOTATATE (Lutathera®)	Yes	Activity (L1)	Optional	EANM [27] MIRD [17]
Bone pain palliation	[^89^Sr]SrCl_2_	Yes	Activity (L1)	Not feasible (L1)	
	[^153^Sm]Sm-EDTMP	Yes	Activity (L1)	Optional	
	[^223^Ra]RaCl_2_ (Xofigo®)	Yes	Activity (L1)	Optional (^99m^Tc/^18^F)	EANM [28] MIRD [14]
Metastatic liver cancer/colorectal	^90^Y-microspheres	Yes	Activity/BSA (L1) Absorbed dose (L2/L3)	Optional advisable(L2)	EANM [29]
	^166^Ho-microspheres	Yes	Absorbed dose (L2/L3)	Required	
Pancreatic adenocarcinoma	^32^P-labelled microparticles	No	Absorbed dose (L3)	Required (volume)	
Radiation synoviorthesis	[^169^Er]Er-citrate [^90^Y]Y-silicate/citrate [^186^Re]Re-sulphide	Yes	Activity (L1)	Not feasible Optional (L1)	EANM [30]
Lymphoma	[^90^Y]Y-ibritumomab tiuxetan (Zevalin®)	Yes	Activity/BW (L1)	Not feasible Optional (^111^In)	EANM [31] MIRD [32]
	[^177^Lu]Lu-lilotomab satetraxetan	No	Activity/BW (L2)	Advisable (L2)	
Metastatic prostate cancer	[^177^Lu]Lu-PSMA-ligands	No/yes after MA^1^	Activity (L1/L2)	Advisable (L2)/optional (L1)^1^	EANM [33]

^1^After marketing authorization by European regulatory authority EMA

*BW* body weight, *BSA* body surface area

## Conclusion

Recommendations are made by the EANM to aid clinical centres in complying to the EC basic safety standards directive. The majority of nuclear medicine therapies can be considered to be standardized, when using an approved radiopharmaceutical or applying an approved device.

Three levels are defined in optimization and prescription of nuclear medicine therapy: (1) activity-based prescription and cohort-averaged dosimetry, (2) activity-based prescription and patient-specific dosimetry and (3) absorbed dose-based patient-specific prescription. In most current clinical treatments, we essentially need to guarantee that the proper activity is administered.

The authors believe that the scheme set out in this position paper will enable the centres to continue progress in the field of nuclear medicine therapy, ensure that new nuclear medicine therapies are introduced clinically and cost-effectively, stimulate research for generating further evidence and will help to optimize and standardize patient-specific therapeutic practices in nuclear medicine in Europe.
